# Evaluation of the accuracy of different apex locators in determining the working length during root canal retreatment

**DOI:** 10.34172/joddd.2020.026

**Published:** 2020-06-17

**Authors:** Pelin Tufenkci, Aylin Kalaycı

**Affiliations:** ^1^Department of Endodontics, Faculty of Dentistry, Hatay Mustafa Kemal University, Hatay, Turkey; ^2^Department of Endodontics, Faculty of Dentistry, Ankara University, Ankara, Turkey

**Keywords:** Electronic apex locator, Retreatment, Working length

## Abstract

**Background.** This study aimed to assess the accuracy of three electronic apex locators (EALs) (Dentaport ZX [J Morita, Tokyo, Japan], Propex Pixi [Dentsply Maillefer, Ballaigues, Switzerland], and iPex II [NSK, Tokyo, Japan]) during root canal retreatment.

**Methods.** The root canal lengths of 90 extracted single-rooted human teeth were determined under a dental operating microscope at ×10 magnification. The actual working length (AWL) was 0.5 mm less than the root length. Electronic measurements were performed with the three EALs. The root canals were instrumented and filled to the actual working length using the lateral compaction technique. After seven days, the teeth were retreated until the retreatment file was applied to the root canal at the working length determined by EALs, and then, the three EALs were used for determining the retreatment working length. Data were analyzed using chi-squared and Kruskal–Wallis tests.

**Results.** In the retreatment, the accuracy of EALs was reported at %83.3 for Dentaport ZX, %83.4 forPropex Pixi, and %80 for iPex II within a tolerance of 0.5± mm of the AWL.

**Conclusion.** Under the limitations of this study, Dentaport ZX, Propex Pixi, and iPex II can be a useful adjunct during retreatment. Clinicians should be aware that residual materials in the root canal during retreatment can affect the accuracy of EALs.

## Introduction

Recurrent infection or re-infection of endodontically treated teeth might require additional treatment procedures. Non-surgical procedures include removing the existing root canal filling, additional preparation, and re-filling of the root canal.^1,2^ In cases of repeated endodontic treatment, it is very crucial to accurately determine the root canal length for removing all the filling materials and debris. The periradicular tissues can be healed by sufficiently enlarging and disinfecting at the accurately determined root canal length.^3^ Bergenholtz et al^4^ reported that only 36% of the teeth with over-filled root canals are treated successfully. They also observed that the root canal treatment’s success increased up to 62% when the working length was accurately determined during the retreatment.

The working length is conventionally determined using a radiographic technique. Due to the anatomic variations and distortions, it is recommended that the radiographic technique should be used along with electronic apex locators (EAL).^5,6^ The use of EALs for working length determination was proposed by Custer^7^ in 1918. The first apex locator was developed by Sunada in 1942, which determined the working length using different resistance values of periodontal ligament and oral mucosa.^8^ Nowadays, apex locators are much more advanced. Root ZX (J Morita, Tokyo, Japan) uses the proportion method by measuring the difference of the impedance values at two different frequencies (0.4 kHz and 8 kHz). Dentaport ZX (J Morita, Tokyo, Japan) is an advanced version of Root ZX, which consists of Root ZX and a rotary motor attached to it.^6^ According to the manufacturers’ recommendations, Propex Pixi (Dentsply Maillefer, Ballaigues, Switzerland) and iPex II (NSK, Tokyo, Japan) are the apex locators that operate with multi-frequency method and are capable of determining the working length in dry or wet canals without calibration. Previous studies have reported that the devices electronically measuring the root canal length had high success rates (83‒100%) in determining the working length.^9-11^

The use of EALs in retreatment is an easy and practical method for determining the working length. According to previous studies, electronic apex locators are not affected by the irrigation solutions used in root canal treatment (sodium hypochlorite, EDTA, chlorhexidine, and normal saline solution).^9-11^ However, according to some reports, the EAL’s accuracy was affected by the presence of debris, organic wastes, calcium hydroxide, gutta-percha, sealer, and solvents.^12-14^ For this reason, the present in vitro study aims to assess the accuracy of Dentaport ZX, Propex Pixi, and iPex II apex locators during root canal retreatment.

## Methods

In the present study, 90 mandibular central and lateral incisors, extracted for periodontal and prosthetic reasons, with no restoration or caries, but with single roots, single canals, and closed apices, were used. All the crowns were cut from the CEJ using a diamond disc to provide a straight reference line for all the measurements. The #15 K file (Dentsply Maillefer) was placed into the root canal until the tip of the file became visible at the foramen under a dental-operating microscope at ×10 magnification (M320 Leica, Leica Microsystems, Wetzlar, Germany). Then, the rubber stopper of the file was completely placed on the pre-flattened root surface and fixed to the file by using flowable composite resin so that the rubber stopper would not slide. The distance between the rubber stopper and the tip of the file was measured with a caliper (Digimatic CD-15DCX; Mitutoyo, Kawasaki, Japan) having 0.01mm sensitivity. The value was calculated by subtracting 0.5 mm from this mean value and named the actual working length (AWL).

The teeth were randomly divided into three groups (n=30) in terms of the electronic apex locator to be used (Dentaport ZX, Propex Pixi, and iPex II). The roots were embedded in alginate. After placing the lip clip of the electronic apex locator in the alginate, the electrode cable’s clips were attached to the #15 K-file to perform the electronic measurements. After completing the setting, the root canals were irrigated with 1 mL of 5.25% NaOCl solution.

Dentaport ZX, Propex Pixi, and iPex II were used according the manufacturers’ instructions. The # 15 K file was advanced in the root canal until the flashing ‘APEX’ bar was indicate on the screen of Dentaport ZX and then withdrawn until the screen showed between ‘APEX’ and ‘1’. For Propex pixi and iPex II, the file was advanced until the ‘0.0’ mark was seen. The values were recorded as the electronic working length (EWL).

### Root canal obturation

After removing the teeth out of the alginate model, the root canals were enlarged with ProTaper (Dentsply Maillefer, Ballaigues, Swiss) rotary files following the order (SX, S1, S2, F1, and F2) at EWL; 5 mL of 17% ethylenediaminetetraacetic acid (EDTA) (Aklar Kimya, Ankara, Turkey), 5 mL of 5.25% NaOCl, and 5 mL of distilled water (final irrigation) were used, respectively. The root canals were dried using paper points (Spident, Nam Dong Kong Don, Inchon, Korea). The teeth were filled at EWL using AHPlus sealer and F2 Protaper gutta-percha cones (Sure-endo, Sure Dent Corp., Korea). The roots in all the groups were stored at 37°C and 100% humidity for 10 days to allow the sealer to set.

### Determining the EWL after removal of root filling (REWL)

In all the groups, the root canal fillings were removed using the ProTaper retreatment file at EWL (not to damage the minor foramen). The D1 was used for removal of the material from the coronal part of the canal, while the D2 and D3 for the removal of the material from the middle and apical third of the canal at 2-Ncm torque and 500 rpm speed. One mL of eucalyptol (Sultan Health-Care, NJ, USA) was dropped into the coronal section of the roots. Then, the solvent penetrated the gutta-percha, and the ProTaper retreatment file was applied to the root canal at the WL determined by EALs. After the last NaOCl irrigation sequence, the attachment clip of the 2-electrode cable of the device was attached to the #25 K-type file to perform the measurements, and the lip clip that was in the alginate was attached to the other electrode. The file was advanced down the root canal until the minor foramen line was specified for EALs, as previously described. The measurements were repeated three times for each tooth, and the mean values were recorded (REWL). Moreover, all the measurements were performed in two hours so that the alginate model would not lose its moisture.

For each tooth, the AWL was extracted from the value measured by the EALs. Positive results indicated measurements longer than the AWL (long measurements), and negative values indicated the measurements shorter than the AWL.

### Statistical analysis

The statistical analyses were performed using SPSS 20.0.1 (SPSS, Chicago, IL, USA). The normality of the variables’ was analyzed using the Shapiro-Wilk test. The differences between the groups were analyzed with Kruskal-Wallis-H tests. The relationships between the groups of normal variables were analyzed using the chi-squared test. The level of significance was set at P<0.05.

## Results

The mean distances from the AWL to file tip for Dentaport ZX, Propex Pixi, and iPex II in treatment and retreatment procedures are presented in Table 1.

**Table 1:** The mean distances between the AWLs obtained by the direct method and the WLs obtained by the electronic methods before root canal preparation and during retreatment

	EWL	REWL
	**Mean ± SD**	**Mean ± SD**
**Dentaport ZX**	0.21±0.33	0.35±0.49
**Propex Pixi**	0.23±0.39	0.33±0.39
**iPex II**	0.23±0.37	0.32±0.47

**Table 2:** Differences between the AWLs and the EWLs of Dentaport ZX, Propex Pixi, and iPex II (before root canal preparation and during retreatment)

Distance from AWL (mm)*	**EWL**						**REWL**					
	**Dentaport ZX**		**Propex Pixi**		**iPex II**		**Dentaport ZX**		**Propex Pixi**		**iPex II**	
	**N**	**%**	**N**	**%**	**N**	**%**	**N**	**%**	**N**	**%**	**N**	**%**
**-1.0 to -0.51**	-	-	-	-	1	3.3	-	-	-	-	1	3.3
**- 0.50 to 0.0**	13	43.3	7	23.3	7	23.3	9	30	8	26.7	6	20
**0.01‒0.5**	14	46.7	19	63.3	19	63.3	16	53.3	17	56.7	18	60
**0.51‒1**	2	6.7	2	6.7	1	3.3	1	3.3	3	10	2	6.7
**<-1.1 and >1.1**	1	3.3	2	6.7	2	6.7	4	6.7	2	6.6	3	10

*Negative values indicate measurements short of the AWL.

After the analysis of data, no statistically significant difference was observed between the devices in terms of the difference between the AWL, which was set to be 0.5 mm shorter than the major foramen, and the values before and after retreatment by using Dentaport ZX, Propex Pixi, and iPex II (P>0.05).

In the retreatment, the accuracy of EALs was 83.3% for Dentaport ZX, 83.4% for Propex Pixi, and 80% for iPex II within a tolerance of ±0.5 mm of the AWL, with 86.6% for Dentaport ZX, 93.4% for Propex Pixi and 90% for iPex II within a tolerance of ±1 mm of the AWL.

## Discussion

In primary endodontic treatments and retreatments, determining the working length is of utmost importance for the root canal preparation and 3D obturation of the root canal.^5^ Brunton et al^15^ reported that too many radiographic images are taken during the root canal retreatment, that the use of radiography solely would be insufficient in determining the working length, and that the patient would be exposed to excessive radiation. Moreover, Alves et al^16^ stated that the radiopacity of root canal filling residuals remaining in the canal during the retreatment might affect the process of determining the working length by using radiography. Therefore, according to previous studies, during retreatment, it is impossible to completely remove the residual materials, sealer, gutta-percha, and solvents from the root canals, which affects the EAL readings.^17,18^

The present study aimed to evaluate the accuracy of three EALs (Dentaport ZX, ProPex Pixi, and iPex II) in the determination of working length during root canal retreatment. EALs operate on electrical principles rather than being dependent on the biological properties of the tissue involved. Therefore, the alginate model established using teeth embedded in the media with electrical resistance similar to the periodontium and identified by Kaufman^10^ was used.

Since the mean distance from the foramen to the apical constriction is approximately 0.5–1.0 mm for all the tooth types,^19,20^ the AWL was determined in the present study by subtracting 0.5 mm from this distance, which the file was seen at the major foramen, as recommended in the previous studies.

In many studies examining the accuracy of EALs, a ±0.5 mm margin was used.^21-23^ It is accepted that the measurements performed at this margin range show a high level of accuracy. Previous studies have shown that the margin range of ±1 mm is more indefinite. One reason for this might be the shape of the enlarging root tip. Moreover, the root canals do not always end with a minor and major diameter at the cemental conjunction base and apical constriction.^24^ Because of such limitations, the ±1 mm margin range is clinically acceptable.

In previous ex vivo studies, no difference was reported between Root ZX, Dentaport ZX, and Root ZX Mini devices.^25^ The accuracy of Root ZX in determining the root canal length by using a model was reported to be 97.37% by Plotino et al^26^ at ±0.5 mm margin range. Aggarwal et al^27^ reported the accuracy of ‘Root ZX’ and ‘Propex’ in determining the working length at 83.3% and 93.3%, respectively, at ±0.5-mm margin range. Puri et al^28^ compared the working length determined by Dentaport ZX and iPex II electronic apex locators with the real root canal length and reported the accuracy at 93.3% for Dentaport ZX and 90% for iPex II at ±0.5-mm margin range. In their in vivo study on Propex Pixi and Root ZX devices, Serna-Pena et al^29^ reported the accuracy at 83.3% for Propex Pixi and 83.3% for Root ZX at ±0.5-mm margin range and 89.9% for Propex Pixi and 100% for Root ZX at ±1-mm margin range. In the present study, the accuracy values were found to be 90% for Dentaport ZX, 86.6% for Propex Pixi, and 86.6% for iPex II at ±0.5-mm margin range and 96.7% for Dentaport ZX, 93.3% for Propex Pixi, and 93.2% for iPex II at ±1-mm margin range. Although there was a similarity between the results, it is thought that the numerical difference might be due to the generation difference between the devices.

Many studies have examined the accuracy of EALs used after retreatment. Goldberg et al^22^ compared the accuracy of three different apex locators (Propex, Root ZX, NovApex) with the real root canal length in determining the working length in root canal retreatment. The researchers reported the accuracy values at 80% at ±0.5-mm margin range and 95% at ±1-mm margin range for Propex, and 95% at ±0.5-mm margin range and 100% at ±1-mm margin range for Root ZX device. In the present study, in comparison with the real root canal length, the accuracy values were reported to be 83.3% for Dentaport ZX and 83.4% for Propex Pixi ±0.5-mm margin range, and 86.6% for Dentaport ZX and 93.4% for Propex Pixi at ±1-mm margin range. The results reported by Goldberg et al^22^ for Propex apex locator are consistent with the present study. However, a comparison of the Dentaport ZX in the present study with those reported in that study, the present results were found to be at lower percentages. Previous studies reported that the residual dentin, debris and root canal filling wastes might affect the results.^12,13^ Uzunoğlu et al^14^ reported that the residual medicaments containing calcium hydroxide, which are applied to the root canals, in the root canals, negatively affect the accuracy of EALs, and this negative effect is directly proportional to the amount of residual debris in the canal. In the present study, it is believed that the lower percentage value of Dentaport ZX might be due to the residual materials in the root canal.

The size of the file used and the apical foramen used have been reported to be important parameters for EALs accuracy.^30,31^ In the present study, no attempt was made to standardize the apical size of the roots because it was not possible to find apically uniform-sized extracted teeth. Uniform-sized apical foramen can be created by widening the roots progressively using bigger instruments, but with this technique, the apical anatomy is changed, and the apically enlarged roots might not mimic the real clinical conditions. In the present study, similar-sized teeth and the same-size instruments were used for all the roots to increase the reliability of the results.

In the present study, in which the accuracy of EAL during the removal of root canal filling was compared to the AWL, no statistically significant difference was observed between the devices but the percentage accuracy of working length determined after the retreatment was found to be lower than the percentage accuracy determined before the root canal treatment. In the retreatment cases, it is recommended that solvents be used for softening and easy removal of gutta-percha.^32^ Er et al^17^ and Ustun et al^33^ reported that the use of a solvent in retreatment procedures might adversely affect the accuracy of EAL devices. The electrical conductivity of solvents could be responsible for the poor accuracy of EALs.

## Conclusion

Under the conditions of this study, Dentaport ZX, Propex Pixi, and iPex II can be a useful adjunct during retreatment. Clinicians should be aware that residual materials in the root canal during retreatment could affect the accuracy of EALs.

## Authors’ Contributions

Concept: PT and AK; design: PT and AK; supervision: A.K; data collection and/or processing: PT; literature search: PT; writing the manuscript: PT and AK.

## Acknowledgments

Not applicable.

## Funding

Not applicable.

## Competing interests

The authors declare no competing interests with regards to the authorship and/or publication of this article.

## Ethics approval

This study was approved by the Research Ethics Committee of Ankara University (30.04.2014-21/16).
